# The impact of translated reminder letters and phone calls on mammography screening booking rates: Two randomised controlled trials

**DOI:** 10.1371/journal.pone.0226610

**Published:** 2020-01-10

**Authors:** Alison Beauchamp, Mohammadreza Mohebbi, Annie Cooper, Vicki Pridmore, Patricia Livingston, Matthew Scanlon, Melissa Davis, Jonathan O’Hara, Richard Osborne

**Affiliations:** 1 Department of Medicine–Western Health, University of Melbourne, Melbourne, Australia; 2 Australian Institute for Musculoskeletal Science (AIMSS), Melbourne, Australia; 3 Faculty of Health, Deakin University, Geelong, Australia; 4 Monash Rural Health, Warragul, Australia; 5 Biostatistics Unit, Faculty of Health, Deakin University, Geelong, Australia; 6 BreastScreen Victoria, Melbourne, Australia; 7 Centre for Global Health and Equity, Faculty of Health, Arts and Design, Swinburne University of Technology, Hawthorn, Australia; Istituto di Ricovero e Cura a Carattere Scientifico Centro di Riferimento Oncologico della Basilicata, ITALY

## Abstract

**Introduction:**

Participation in mammographic screening for breast cancer in Australia is approximately 54% among the general population, but screening among women from some culturally and linguistically diverse (CALD) backgrounds is lower. BreastScreen Victoria apply strategies to increase screening including reminder letters and phone calls; however, these are usually provided in English. Using intervention strategies generated from the Ophelia (OPtimise HEalth LIteracy and Access) community co-design process, translated mammography reminder letters and in-language phone calls were tested within two randomised control trials (RCTs).

**Methods and analysis:**

Women aged 50–75 years who were due for their 2-yearly screening mammography (for RCT#1) or were under-screened, i.e. ≥27 months since last screen (for RCT#2) were randomised into intervention or control groups. RCT#1 compared sending women routine reminder letters (English only) with translated (Arabic or Italian) letters. RCT#2 compared reminder telephone calls to women in their preferred language (Arabic or Italian) to no telephone call. The primary outcome for each trial was screening booking rates within 14-days. Primary outcomes were tested using Pearson’s chi-square test. Rates within language group (incidence ratio: IR) were compared using the Cochran-Mantel-Haenszel test.

**Results:**

For RCT#1 (letters) 1,032 women were randomised into the intervention arm or to usual care. Uptake of screening bookings was similar between both groups, with no differences observed by language group. For RCT#2 (phone calls), 195 women were randomised to the intervention group or to usual care. Overall, 64.2% of women in the intervention arm and 6% in the control arm booked a screening appointment within 14 days (p<0.0001). The IR (95%CI) of booking was 10.1 (3.9, 26.3) times higher among Italian women, and 11.6 (2.9, 46.5) times higher among Arabic women in the intervention compared to usual care groups.

**Discussion and conclusion:**

A service improvement initiative derived from community members and breast screen providers was found to be highly effective. This evidence informed the service provider, BreastScreen Victoria, who have implemented these improvements into routine practice to improve screening among CALD groups and reduce health inequalities.

## Introduction

Globally, breast cancer is the most common cancer in women. In Australia, 16,753 new cases of breast cancer were diagnosed in 2014, and it was the second most common cause of cancer-related death among females [1]. National population-based mammography screening programs for early detection of breast cancer are established in most developed countries, and have been associated with mortality reductions of 15–30% in women aged 50–74 years [2–6]. Although shown to be effective in reducing mortality, mammography screening programs are not without controversy [7, 8]. The importance of providing information about the benefits and harms has been highlighted so that women can make an informed decision about their choice to screen or not [9].

In Australia, mammography screening and any follow-up assessments for abnormal results are provided free of charge under the auspices of BreastScreen Australia, the national breast cancer screening program [10]. The current recommendation from BreastScreen Australia is that biennial screening mammography is undertaken for women aged 50 to 74 years [11]. BreastScreen Australia specifically targets women in this age group using electoral roll and Medicare data to contact women.

In countries with national mammography screening programs, participation rates range between 20% in Turkey to over 80% in Japan [12]. Participation rates in Australia are approximately 54% nationally [10]. indicating that despite access to free screening, a significant proportion of women are not attending. Population subgroups at greater risk of not screening include women from some culturally diverse groups and those from lower socioeconomic backgrounds [13]. Generally, rates of cancer screening for migrants to Australia are slightly lower than the Australian born population [14–16]. There is also some evidence of a socioeconomic gradient in screening [17–19], and while this is not conclusive [20], studies have identified patterns of more advanced disease at diagnosis among diverse cultural or lower socioeconomic groups [21–23]. It is therefore important to target strategies to improve uptake of screening among these at-risk groups.

In Victoria, Australia, the north west area of Melbourne is considered a priority area for increased screening by BreastScreen Victoria (BSV; an accredited part of BreastScreen Australia). Although participation rates in the region are similar to state-wide rates, there are sub-regions with much lower rates of screening, and areas of marked socioeconomic disadvantage. The region is culturally and linguistically diverse (CALD) with large Italian and Arabic communities. Rates of screening among these communities tend to be lower than other women in the region, representing a significant proportion of culturally diverse women who are under-screened; i.e. not undergoing regular 2-yearly mammography screening. The motivation for this study was recognition by State Government and BSV that innovative strategies were required to improve service provision in the region. This present study forms part of the Ophelia BreastScreen study, in which health literacy-based interventions were co-designed with community members and breast screen providers to improve the acceptability and accessibility of the screening program to Italian, Arabic, and Aboriginal and Torres Strait Islander women in the North-West screening region [24, 25].

Within large national and state-wide organisations that conduct screening mammography programs, implementation of new interventions to improve participation among cultural groups can be challenging. A cost-effective approach may be to tailor existing processes to be more culturally appropriate. One such process is that of reminder prompts for women to attend breast screening. In Victoria, routine reminder letters in English are sent to women who are due for their repeat bi-annual screen by BSV. On occasion, reminder phone calls in English are also made for women who are considered to be “lapsed screeners”, defined as being more than 27 months since their last screening mammography. These routine reminder letters and phone calls are shown in other studies to be effective at increasing uptake of screening, and are considered among the most successful strategies for recruitment to mammography screening [26–28]. Despite their effectiveness in the general population, there is little evaluation of their impact upon women from CALD communities. Systematic reviews have examined the impact of interventions to increase cancer screening among African-American minority groups in North America [29], Arabic women in Qatar and North America [30], and among Asian women living in Western countries [31]. However, few previous studies have examined the impact of providing translated routine reminder letters or phone calls among CALD communities [32–34]. This represents an important gap in the evidence for strategies to increase the uptake of mammography screening among culturally and linguistically diverse women.

This paper describes two randomised controlled trials (RCTs) to evaluate the effect of providing translated mammography screening routine reminder letters or phone calls for Arabic and Italian women living in North West Melbourne. Two RCTs were combined in this study as they were conducted at the same time, among women in the same catchment areas of Melbourne, and involve the same stakeholder groups. The study was informed by the Consort Statement (2010) [35].

### Objectives

1. RCT#1 (letters) aimed to assess the impact of providing translated routine reminder letters on rates of booking for mammography screening within 14 days of the letter being sent among Arabic and Italian women.
2. RCT #2 (phone calls) aimed to assess the impact of in-language reminder phone calls to lapsed screeners on rates of booking for mammography screening within 14 days of the phone call among Arabic and Italian women.

It was hypothesised that there would be a higher mammography booking rate among women who received reminder letters in their preferred language compared to women who received usual care. It was also hypothesised that there would be a higher booking rate among women who were lapsed screeners and received a reminder phone call in their preferred language compared to women who received usual care.

## Methods

### Study design

Two independent single blinded, two-armed, RCTs were conducted. In RCT #1 (letters), the intervention consisted of sending routine reminder letters, translated into a woman’s preferred language spoken at home (Arabic or Italian) as recorded at her last breast screen attendance. The control arm consisted of usual care, whereby the reminder letters were sent in English. In RCT #2 (phone calls), the intervention arm consisted of telephoning women who were “lapsed screeners” in their preferred language (Arabic or Italian). The control arm consisted of usual care, in this instance no phone calls.

**Fig 1:**
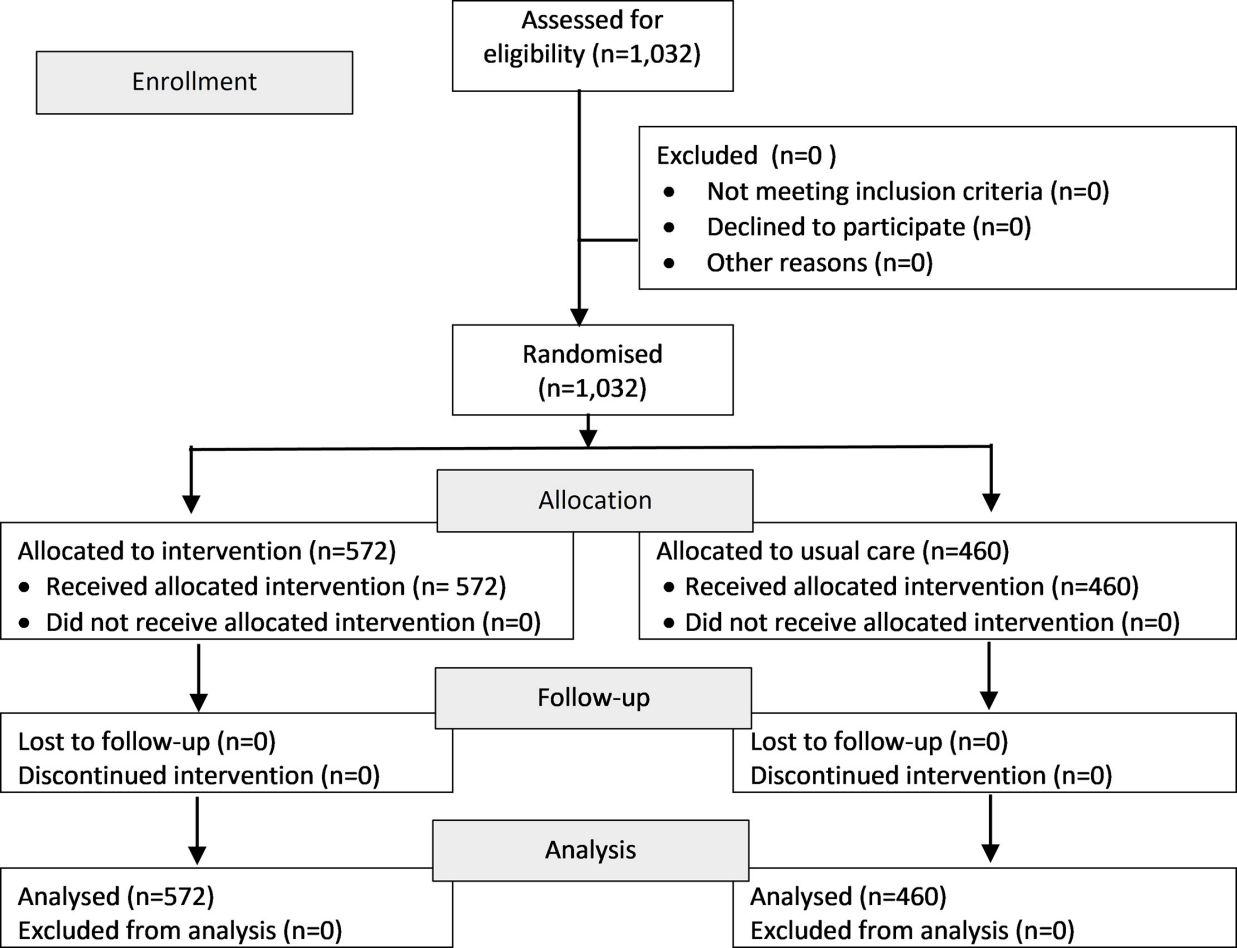
CONSORT flow diagram for letters trial [41].

**Fig 2:**
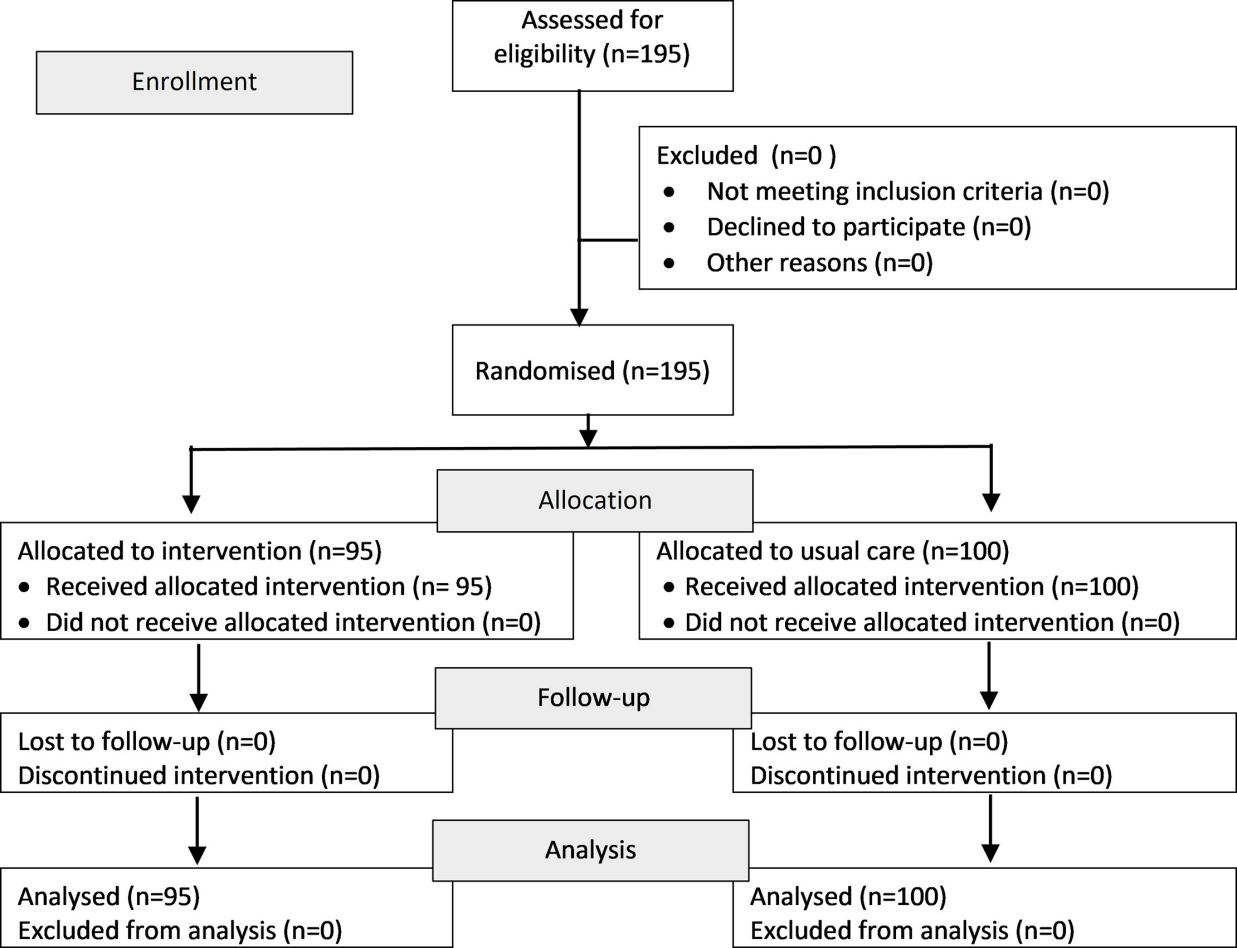
CONSORT flow diagram for phone calls trial [41].

The study was designed to adhere to the Good Clinical Practice (GCP) Guidelines for National Ethics Standards and followed the RCT CONSORT statement in reporting, ensuring it meets international ethical and scientific quality standards for reporting clinical trials (Figs 1 and 2) [35]. This study was registered with the Australia and New Zealand Clinical Trials Registry, Trial Registration Number ACTRN12617001163392.

### Study setting

This study took place in partnership with BreastScreen Victoria and recruited women from North West Melbourne, Australia during 2017.

### Sample/Participants

The sample comprised all women aged 50–75 years who were due for their 2-yearly screening mammography or were lapsed screeners (i.e. >27 months since their last screen), and who lived within the North-West Melbourne catchment area or whose preferred BreastScreen clinic was based in that catchment area. During the recruitment period (July to November 2017) there were an estimated 1,077 Italian-speaking women and 416 Arabic-speaking women in North-West Melbourne who met the eligibility criteria for RCT #1 (letters). For RCT #2 (phone calls), the most recent data available from BSV for lapsed screeners by language groups showed that in 2014, there were 243 Arabic women and 553 Italian women in Victoria who met the eligibility criteria [36].

### Eligibility criteria

Inclusion criteria were: 1) all women who lived in the BSV screening region of North West Melbourne or whose preferred clinic was in that same region; 2) aged 50–74 years; 3) had previously identified on their BreastScreen registration form that Italian or Arabic was their preferred language spoken at home, and; 4) for RCT #1 (letters) were due to receive their 2-yearly routine reminder letter, or; 5) for RCT #2 (phone calls) had not re-screened for at least 27 months. Women who had been discharged from the BSV program were excluded from this study (reasons for discharge include death, a diagnosis of breast cancer, or a request to be discharged from the program).

### Pre-randomisation

Women were not individually recruited into the study. Participants were selected from an administrative dataset using an automated procedure, based on their eligibility for participation as described above, between July and December 2017. For RCT1, women who were due for a reminder letter within the next month were identified and randomised on at least a monthly basis for the duration of the study period. For RCT2, identification and randomisation occurred near the beginning of the study period, and the intervention commenced approximately three weeks later. The two trials were discrete and there was no overlap: women in RCT2 were identified at the start of the study period and were therefore > = 27months since their last screen; women in RCT1 were identified on a continuing basis from the start of the study period and were screened < = 27months previously.

### Randomisation

Individual women were the unit of randomisation. Randomisation to intervention or control groups was undertaken using a 1:1 ratio, stratified by language group (Italian and Arabic). Within each stratification, an automated randomisation procedure using a random number generator was used for random allocation of women into each arm. In order to maintain random allocation concealment, there was no direct contact at any stage between the generator and executors of the random assignment and the study research team.

### Sample size

For RCT #1 (letters), for a pooled sample of Italian and Arabic participants, the aim was to detect a 10% difference in booking rates between the intervention and the usual care group; a pooled sample size of 373 per intervention arm was considered sufficient to detect this difference (based on 80% power, a Type 1 error rate of 5% and a baseline 14 day booking rate of 53.2%). A sub-group analysis by language group (i.e. Italian and Arabic) was also planned, and as the number of Italian women due to be rescreened was approximately 30% higher than for Arabic women, this sample size was stratified as:

- Arabic women—160 in each arm to detect a 15% increase in booking rates (from a baseline screening rate of 55.7% (36)). Based on findings from other studies [30, 37], it was reasonable to assume that this slightly higher response rate was achievable for Arabic women.
- Italian women—213 in each arm to detect a 13.5% increase in booking rates (from a baseline screening rate of 45.4%) [36].

For RCT #2 (phone call), lapsed screeners were randomised to intervention and usual care. Assuming a conservative current booking rate at 14 days of 10% for lapsed screeners [36], a 15% increase in booking rate (power = 80% and Type 1 error rate = 5%) would require a total pooled sample size of 200 Italian and Arabic participants. Given that the number of Italian women was approximately 30% higher, this sample size was stratified as:

- Arabic women—43 in each arm. This would detect a 25% or more increase in booking rates.
- Italian women—57 in each arm. This would detect a 20% or more increase in booking rates.

The sample sizes were not inflated to allow for drop out as not booking a breast screen within the defined time interval was defined as unsuccessful screening, in accordance with the intention-to-treat (ITT) principle. Administrative data only were used, and as such, it was not feasible to collect data about why a participant did not attend for screening.

### Blinding

All research staff, including the study biostatistician were blinded to group allocation. Success of blinding was assessed at study end using the Blinding Index [38]. Although AC was aware of the allocation, the remaining investigators were unaware of the comparator. Blinding of participants was not possible due to the nature of the interventions, and participants were not informed about the trial aims. MM, AB and JO analysed the data, and were all blinded to group allocation.

### Interventions

The concept for both interventions arose from the Ophelia BreastScreen study [24], in which ideas for service improvement initiatives to increase screening had previously been generated by BreastScreen providers and women from the Arabic and Italian communities in North-West Melbourne, and staff from the BSV Coordination Unit. Eight interventions were subsequently co-designed and evaluated, including the two RCTs presented here.

#### RCT #1

As part of their core business, BSV sends routine reminder letters to women who are due for their bi-annual screen, using automated printing and mailing processes. *Group 1*. *Reminder letters in language*: This group received a single routine reminder letter in their preferred language spoken at home, as recorded by BSV at their last breast screen. Within the same envelope, the invitation in English was also provided. We included letters in the woman’s preferred language and in English as we did not assume that all women can read and so may have required someone to read the letter to them. The letter in English was not altered in any way. The text of the translated letter varied slightly from the usual reminder letter, containing brief, simplified information about the purpose of screening, information about how to book a screen, and contact details for BSV. Letters were translated by a professional translation service, and also included a photo and quote from an Italian or Arabic GP. The content of the letter was based on findings from the earlier Ophelia BreastScreen study and readability was tested by a community member from each language group. *Group 2*. *Usual care*: This group received a routine reminder letter in English, containing information about booking, statistics about the risk of breast cancer and the purpose for screening. On the back of the letter, information on how to request an interpreter was provided in the most commonly spoken languages (including Arabic and Italian).

#### RCT #2

Group 1. Reminder phone call. This group received a telephone call in their preferred language to remind and assist them to book a screening appointment. The phone number provided by the woman at her last breast screening appointment was used. Each phone call was expected to last between 4–8 minutes and was made by one fluent speaker each of Arabic and one of Italian, each of whom was able to switch to speaking English if the woman preferred. The two outbound callers were provided with training and a script to follow, and were able to book a screening appointment for participants whilst on the telephone. The standard telephone script used by BSV for ad hoc reminder phone calls was translated into both Arabic and Italian, seeking to ensure the content in each language version was similar. The content of the script included an introduction by the caller and the purpose of the call. Where requested by a woman, the caller was able to make a booking during the phone call, and advice provided about where attend for screening. Callers could deviate from the script if required and were provided with a list of common questions and answers. A maximum of three attempts was made to contact each woman. We did not leave voice messages due to privacy.

Group 2. Usual care. This group received usual care, consisting of no reminder phone call.

### Outcomes

The primary outcome from both RCTs was rates of booking a screening appointment within 14 days. This time frame was selected as women who do not respond to their first reminder letter within 14 days automatically receive a second reminder letter in English. For consistency between the two RCTs, 14 days from the time of the phone call was also used for the phone calls trial.

### Governance

BSV was the governance body for this trial. Data were collected by BSV and then provided to the research team in a non-identifiable format on completion of the data collection period, using a password protected spreadsheet.

### Analysis

All analyses were conducted in accordance with the International Conference on Harmonization E9 statistical principles [39], and reported according to the CONSORT recommendations [40, 41].

For each RCT, the preferred and secondary outcome analyses were based on all randomised participants (intention-to treat principle). Testing absolute difference for two independent proportions using the Z-test with a 2-sided alpha were considered for the sample size calculation. Outcomes were presented as frequencies and percentages for each study arm. Differences in booking rates between control and intervention groups were examined using Pearson’s chi-square test. Pre-planned secondary analysis on language group was undertaken using the Cochran-Mantel-Haenszel test followed by sub-group analyses based on language status. Incidence ratios (IR) with the corresponding 95% confidence intervals (CIs) were reported, as were risk difference and 95% CIs where appropriate. The Cornfield approximate method was used for estimating confidence intervals [42]. A p-value of <0.05 (two-sided) was considered statistically significant. No adjustment for multiple comparisons was implemented for the pre-planned sub-group analyses. Data were analysed using Stata version 15.

### Ethical considerations and dissemination

This study was approved by the Deakin University Human Research Ethics Committee (DUHREC, #2015–317). Individual consent was not sought from participants as this intervention was a service improvement initiative undertaken by BreastScreen Victoria. This consent waiver was approved by DUHREC.

## Results

As shown in Fig 1, for RCT#1 (letters) a total of 1,032 women were randomised to usual care (n = 460) or to receive the intervention (n = 572). This number was higher than the original proposed sample size of 746 because it took longer to reach the required numbers of Arabic compared to Italian women. Information about returned mailings for the study sample in RCT#1 was not collected, although the overall rate of returned mailings for routine reminder letters for the time period was 2%.

Table 1 describes the outcome frequency and proportion of participants in each trial arm. A total of 38.9% of women in the usual care group and 37.4% in the intervention group booked a screening appointment within 14 days of the mail out. The difference was not statistically significant. There was no difference in the incidence rate of appointment bookings (1.5%, 95%CI: -7.5%, 4.5%).

**Table 1:** Frequency and proportion of booking a screening appointment within 14 days in the letters trial.

	Usual care	Intervention	Total
Did not book	281 (61.1%)	358 (62.6%)	639 (61.9%)
Booked	179 (38.9%)	214 (37.4%)	393 (38.1%)
(total)	460	572	1,032

There were 322 Arabic women and 710 Italian women participating in the letters trial (Table 2). In the intervention group, 29.9% of Arabic women, and 40.4% of Italian women booked a screening appointment within 14 days of the letter being sent. In the usual care group, 30.4% of Arabic women and 43.4% of Italian women booked an appointment.

**Table 2:** Frequency and proportion of booking a screening appointment within 14 days in the letters trial by language group.

	Usual care	Intervention	Total	p-value*
***Arabic women***				
Did not book	110 (69.6%)	115 (70.1%)	225 (69.9%)	
Booked	48 (30.4%)	49 (29.9%)	97 (30.1%)	
(total, Arabic women)	158	164	322	0.922
***Italian women***				
Did not book	171 (56.6%)	243 (59.6%)	414 (58.3%)	
Booked	131 (43.4%)	165 (40.4%)	296 (41.7%)	
(total, Italian women)	302	408	710	0.433

*using Pearson’s chi-square test

Sub-group analyses showed no significant difference between intervention and usual care groups for either Arabic or Italian women (Table 3). Tests for homogeneity showed no significant interaction between language group and the intervention, hence both language groups were combined to calculate a common IR. Similar to the pooled incidence ratio, the combined incidence ratio using the Cochran-Mantel-Haenszel method showed no difference (IR 0.94; 95% CI: 0.81, 1.10).

**Table 3:** Comparison of screening appointment booking rates according to cultural group in the letters trial: Cochran-Mantel-Haenszel test and sub-group analysis.

	Incidence ratio^1^	95% CI	p-value
Arabic women	0.98	0.71, 1.37	>0.999^2^
Italian women	0.93	0.78, 1.11	0.442^2^
C-M-H combined	0.94^3^	0.81, 1.10	0.458^4^

^1^ Control group as reference category

^2^ Fisher’s exact test for sub-group analysis

^3^ Cochran-Mantel-Haenszel combined IR

^4^ Test of homogeneity (interaction between cultural group and intervention)

For RCT#2 (phone calls), a total of 195 women were randomised to the intervention group (n = 95) or usual care (n = 100). We were unable to recruit the planned 200 women given an underestimation of the projected number of Arabic and Italian women who would be >27months since their last breast screen within the study time period. Due to time and resource limitations, the trial was closed at 195 participants. The allocation of women to groups is shown in Fig 2.

Table 4 describes the outcome frequency and proportions in the trial arms. A total of 64.2% (61 women) in the intervention arm and 6% (n = 6) in the control arm booked a screening appointment within 14 days of the phone call. This difference was statistically significant (Pearson’s chi-square test p <0.0001). There was a 58.2% (95% CI: 47.5%, 68.9%) difference in the incidence rate of appointment booking (usual care vs. intervention). This was equivalent to an incidence ratio of 10.7 (95% CI: 4.9, 23.6), showing the intervention group was 10.7 times more likely to make a booking within 14 days of receiving a reminder phone call compared to the usual care group.

**Table 4:** Frequency and proportion of booking a screening appointment within 14 days in the phone call trial.

	Usual care	Intervention	Total
Did not book	94 (94.0%)	34 (35.8%)	128 (65.6%)
Booked	6 (6.0%)	61 (64.2%)	67 (34.4%)
(total)	100	95	195

A total of 80 Arabic women and 115 Italian women participated in the phone trial (Table 5). Of these, 54.1% of Arabic women in the intervention group and 70.7% of Italian women in the intervention group booked a screening appointment within 14 days of the phone call. This compares to 4.7% for Arabic women and 7.0% for Italian women in the usual care group.

**Table 5:** Frequency and proportion of booking a screening appointment within 14 days in the phone call trial by language group.

	*Usual care*	*Intervention*	*Total*	p-value*
***Arabic women***				
*Did not book*	*41 (95*.*4%)*	*17 (46*.*0%)*	*58 (72*.*5%)*	
*Booked*	*2 (4*.*7%)*	*20 (54*.*1%)*	*22 (27*.*5%)*	
*(total*, *Arabic women)*	*43*	*37*	*80*	*<0*.*0001*
***Italian women***				
*Did not book*	*53 (93%)*	*17 (29*.*3%)*	*70 (60*.*9%)*	
*Booked*	*4 (7*.*0%)*	*41 (70*.*7%)*	*45 (39*.*1%)*	
*(total*, *Italian women)*	*57*	*58*	*115*	*<0*.*0001*

*using Pearson’s chi-square test

Sub-group analyses showed in both language groups the differences in booking rates were statistically significant (Table 6). The rate of booking was 10.1 times higher among Italian women, and 11.6 times higher among Arabic women in the intervention compared to the usual care group. Tests for homogeneity showed no significant interaction between language group and intervention, hence both language groups were combined to calculate a common IR. Similar to the pooled IR (Table 4), the combined IR using the Cochran-Mantel-Haenszel method showed a 10.6 increase in the booking rate in favour of the intervention group.

**Table 6:** Comparison of screening appointment booking rates according to language group in the phone call trial: Cochran-Mantel-Haenszel (CMH) test and sub-group analysis.

	Incidence ratio^1^	95% CI	p-value
Arabic women	11.6	2.9, 46.5	<0.0001^2^
Italian women	10.1	3.9, 26.3	<0.0001^2^
C-M-H combined	10.6^3^	4.8, 23.2	0.8679^4^

^1^ Control group as reference category

^2^ Fisher’s exact test for sub-group analysis

^3^ Cochran-Mantel-Haenszel combined IR

^4^ Test of homogeneity (interaction between language group and intervention).

## Discussion

Breast cancer screening rates among some culturally and linguistically diverse groups are lower than the general population [14–16]. BreastScreen Victoria sought to develop and test strategies to engage Italian and Arabic speaking women using letters and phone calls in their preferred language, and compare these service innovations with usual practice. In controlled trials, sending letters in preferred language showed no difference to letters in English. However, making a telephone call to women in their preferred language was over 10 times more effective than usual care. This simple service improvement, derived from a community co-design process (Ophelia), has led to BreastScreen Victoria changing a service provision practice, and has the potential to improve screening rates among diverse minority language groups.

Routine reminder letters have been shown to be effective at prompting women to book a screen [26–28] including among lower socioeconomic populations [43]. There is less consistent evidence for their effectiveness in culturally diverse groups [29]. although one qualitative study identified health reminders as a preferred approach for ethnic minorities [44]. In our study, the translation and cultural tailoring of the invitation message did not appear to make a difference, and it may be that even more specific targeting is required. Of note, the overall booking rate of 30.4% for Arabic women in the usual care group of RCT1 was lower than the baseline rate of 55.7% obtained from population statistics for Arabic women in North West Melbourne 2014–2016. Reasons for this difference are not clear, although the different time periods for obtaining these rates may have impacted on the variation seen.

Phone calls in a woman’s preferred language may engage her in the first crucial moments of a ‘cold call’. This strategy appears to be a relatively low-cost intervention with a high success rate, and BSV are planning to expand this practice to other language groups over time. Other studies using reminder calls have seen similar results to ours [32, 45–47], although some provided more than one call [34, 48], and others used a tailored counselling-based approach rather than a simple reminder [45, 49]. There are few studies which examined whether cultural or linguistic tailoring of the telephone call has an added impact although in studies where this is considered, the effectiveness of telephone calls remains high. In an inner city area in the UK with high ethnic diversity, an observational study of over 10,000 women found reminder calls in multiple languages following an initial invitation letter substantially improved breast cancer screening uptake (67% vs. an expected 57%) [32]. An earlier study using periodic reminder telephone calls over an 18-month period for Spanish or English-speaking women for breast, cervical or bowel cancer screening in combination with printed educational materials found that Spanish-speaking women receiving the intervention were more likely to be up-to-date with breast cancer screening than English-speaking women (adjusted OR 2.28 (1.38–3.77) vs. 1.32 (0.73–2.40)) [34]. In a third study, reminder phone calls in Spanish or English in combination with a reminder postcard were compared to a reminder postcard only. Results by language were not presented, but compared to those receiving the postcard only, women receiving the phone call were almost twice as likely to attend screening within 30 days (adjusted OR 1.91, 1.37–2.65) [50]. In summary, while a reminder telephone call may contribute the most to a woman’s decision to screen, the limited evidence suggests that translation of the message remains beneficial.

### Strengths and limitations of this study

Our study contributes to the limited evidence about the impact of translated reminder letters and phone calls on rates of booking or screening for breast cancer screening. Population-based RCTs with individual randomization are the gold standard for evaluating interventions, and this study reflects real-world implementation. Participants were selected from two language groups in one area of Melbourne only and findings may not be generalizable to other regions or to other language groups. Further investigation of the mechanisms underlying the effectiveness of reminder phone calls in language is warranted; in particular, whether it is the cultural aspects of this intervention or simply the phone call that creates the effect. A standard telephone script was used for both Arabic and Italian women, and this may have limited the ability to address specific cultural barriers to screening. We were unable to collect data on the number of call attempts or whether telephone numbers had changed. Further, there were some differences in anticipated and actual sample sizes. For RCT1, due to the automated nature of the mailing system, we were unable to stop accruing participants to the Italian subgroup once the original proposed sample size was reached. This technical issue was unforeseen at the time of starting the intervention. The increase in sample size was accepted by the approving ethics committee. For RCT2, we recruited 195 of the 200 women, based on sample size calculations. This is unlikely to have affected the results as a clear effect was identified. Finally, booking an appointment does not necessarily mean that a woman attended that appointment.

## Conclusion

Using the Ophelia co-design approach, BreastScreen providers and women from the Arabic and Italian communities generated strategies to enhance the cultural relevance of reminder letters and phone calls. During routine service provision, robust evidence was generated to inform the service provider (BSV), who have implemented improvements into routine practice to increase screening among CALD groups.

## Supporting information

S1 Checklist CONSORT checklist. (DOC)

S1 File Data_Phone calls. (CSV)

S2 File Data_Letters. (CSV)

S3 File Data dictionary. (DOCX)

S1 Protocol Study protocol. (PDF)

S2 Protocol Study protocol description. (PDF)
