# Supplementary material for: The impact of translated reminder letters and phone calls on mammography screening booking rates: Two randomised controlled trials
Source: PLoS One. 2020 Jan 10;15(1):e0226610. doi: 10.1371/journal.pone.0226610 (PMC6953872; doi:10.1371/journal.pone.0226610)
Supplement: S3 File — (DOCX) [file pone.0226610.s004.docx]

| **PONE-D-19-04032_Data dictionary** | |
| --- | --- |
|  | |
| **Phone trial** | |
| **Variable name** | **Labels** |
| culturalgroup | 1=Arabic  2=Italian |
| intervention | 1=Usual care  2=Phone call in language |
| OUTCOME_Y | 0=Did not book within 14 days  1=Booked within 14 days |
| CULTURALGROUP_1 | 0=Italian  1=Arabic |
| CULTURALGROUP_2 | 0=Arabic  1=Italian |
| INTERVENTION_1 | 0=Usual care  1= Phone call in language |
| INTERVENTION_2 | 0= Phone call in language  1=Usual care |

| **Letters in language trial** | |
| --- | --- |
| **Variable name** | **Labels** |
| culturalgroup | 1=Arabic  2=Italian |
| intervention | 1=Letter in language  2=Usual care |
| OUTCOME_Y | 0=Did not book within 14 days  1=Booked within 14 days |
| CULTURALGROUP_1 | 0=Italian  1=Arabic |
| CULTURALGROUP_2 | 0=Arabic  1=Italian |
| INTERVENTION_1 | 0=Usual care  1= Letter in language |
| INTERVENTION_2 | 0= Letter in language  1=Usual care |
