# Supplementary material for: The impact of translated reminder letters and phone calls on mammography screening booking rates: Two randomised controlled trials
Source: PLoS One. 2020 Jan 10;15(1):e0226610. doi: 10.1371/journal.pone.0226610 (PMC6953872; doi:10.1371/journal.pone.0226610)
Supplement: S1 Protocol — (PDF) [file pone.0226610.s005.pdf]

Optimising breast screening in Melbourne's North West project (Ophelia BreastScreen Project): Evaluation of intervention 1.1 - letters and phone calls in language

1) Background to the project

The project was developed to identify the health literacy strengths and challenges associated with women's engagement with breast screening in North-west Melbourne and to generate and test solutions to identified barriers to screening. Particular groups of interest are women from the Italian, Arabic and Aboriginal and Torres Strait Islander communities.

2) Progress to date

The Health Literacy Questionnaire (HLQ) was administered to 429 women in North-west Melbourne. Respondents included 139 women from the broader community, 172 women from the Italian community, 73 women from the Arabic community and 45 from the Aboriginal and Torres Strait Islander community. Interviews were also conducted with 21 women.

Survey results were used to identify clusters of women within each cultural group with differing patterns of health literacy strengths and needs. These clusters were then explored in depth across ten separate workshops with BreastScreen Victoria (BSV) service providers, women from the broader community, the Italian community, and the Arabic community.

In each workshop, participants discussed the main issues identified in relation to women's breast screening participation. Two yarning circles were also held with Aboriginal and Torres Strait Islander women where they told stories about their experiences of breast screening. Overall, 321 ideas for strategies to improve access and engagement with breast screening were generated.

A further workshop was then held, comprising BSV staff, community partners, and policy leads from the Department of Health and Human Services, in order to identify which ideas could be further developed to. Deakin and BSV subsequently collated findings from all workshops and the yarning circles, leading to a proposed intervention plan to improve uptake of breast screening.

3) Intervention Plan

Seven key interventions were proposed by BSV, as follows:

1. Provide reminder letters +/- reminder phone calls in women's preferred language (Arabic or Italian). This will be a randomised controlled trial.
2. Disseminate messages about breast screening via Arabic and Italian community radio and newspapers
3. Develop a pictorial guide to illustrate what happens during screening.
4. Provide training to North West clinic staff in cultural sensitivity and customer service
5. Implement the Cancer Council Victoria's Peer Education Program
6. Engage with local pharmacies to deliver breast screening messages
7. Commission an Aboriginal health or community service to trial customized capes

BSV will independently implement each of these interventions as service improvement initiatives. Deakin is not involved in implementation of these initiatives. Deakin's role will now be to evaluate these interventions and any advice provided to BSV relates to evaluation activities only.

We will seek ethics modification to conduct evaluation of each intervention as BSV finalises its plans for that intervention. This current modification seeks approval to conduct evaluation of **intervention 1** only. The following section details the approach to evaluation of this intervention.

Intervention 1. BreastScreen Victoria will provide reminder letters +/- reminder phone calls in women's preferred language (Arabic or Italian).

Background: Women who are due for their 2-yearly screening mammogram are routinely sent a letter by BreastScreen Victoria (BSV) to invite them to re-screen. On an ad hoc basis, reminder phone calls are also made to women who remain overdue for their 2-yearly screen, despite having received a reminder letter("lapsed screeners"). The reminder letters and phone calls are currently in English. Findings from our needs assessment indicate that women from Arabic and Italian communities do not always understand the purpose of the letter or phone call because they are in English. When women attend for

screening the first time, they are asked to identify their preferred language, and this information is held by BSV.

Aim: To evaluate whether sending reminder letters and/ or reminder phone calls in a woman's preferred language (Arabic or Italian) is associated with an increase in rates of booking a breast screening appointment ("booking rates").

Please note: The intervention will be delivered by BSV as a service improvement initiative. Deakin researchers will not be involved in delivery of this intervention, but we are seeking ethics approval to evaluate the outcomes. All data will be non-identifiable to Deakin researchers, and consent from individual women will not be sought.

Study design: A randomised controlled trial will be conducted. Individual women will be the unit of randomisation (see **Figure 1**).

Sample: The sampling frame will be all women who live in the BSV screening region of North-west Melbourne who are eligible for screening and who have previously identified their preferred language as Italian or Arabic, and who: 1) are due to receive their 2-yearly routine reminder letter, or: 2) have not re-screened despite having received a routine reminder letter at least 3 months ago. ***Exclusion criteria:*** women who do not indicate Italian or Arabic as their preferred language; women living outside the North West Melbourne catchment area; women not eligible for screening.

Intervention: Routine reminder letters +/- phone call in preferred language. *Group 1. Reminder letters in language:* This group will receive a single routine reminder letter in their preferred language (as recorded by BSV at their initial breast screen) on one side of the letter and English on the other.

Group 2. Reminder letter and phone call in language: This group will receive a single routine reminder letter in their preferred language as for Group 1, and one follow-up reminder phone call if they have not booked a screening appointment within 2 weeks of the reminder letter being posted.

Group 3. Usual care: This group will receive a routine reminder letter in English.

Intervention: Follow up phone call for "lapsed screeners". *Group 1. Reminder phone call.* This group will receive a telephone call in their preferred language to remind and assist them to book a screening appointment.

Group 2. Usual care. This group will receive usual care, which currently consists of ad hoc phone calls made in English by BSV when particular screening sites have low rates of attendance.

Main outcome: The primary outcome from this study will be rates of booking a screening appointment. The secondary outcome will be the source of referral as self-reported by women in the study if and when they book a screening appointment.

Evaluation procedure: BSV will provide the following non-identifiable data to Deakin researchers for evaluation of this intervention: Outcomes of women in each intervention arm (booked screening within 6 weeks vs. did not book screening); preferred language of each woman; screening site; source of referral as reported by women when they book a screening appointment.

Randomisation: BSV has a procedure built into their database software that allows for random allocation of women to each arm. This process has been used previously by BSV, has inbuilt quality control processes, and is their preferred method of randomisation.

Sample size routine recall intervention women **(See Figure 1.)** For a pooled sample, the aim is to detect a 10% difference in screening rates between each intervention and the usual care group; a pooled sample size of 373 per intervention arm will be sufficient to detect this difference (based on 80% power and a Type 1 error rate of 5%). We are interested in sub-group analysis by cultural group, and as the number of Italian women due to be rescreened is approximately 30% higher than for Arabic women, this sample size can be randomly allocated as:

- Arabic women=160 in each arm. This will detect a 15% increase in screening rates (from a baseline screening rate of 56%). Based on findings from other studies, it is reasonable to assume that this slightly higher response rate is achievable for Arabic women

- Italian women=213 in each arm. This will detect a 10-15% increase in screening rates.

Given the nature of the study design, there is no need to inflate the sample sizes to allow for drop out. A sequential sampling method will be appropriate as each women is identified by a unique case number

Sample size lapsed screeners Lapsed screeners will be randomised to intervention and usual care. Assuming a conservative current screening rate of 10% for lapsed screeners (this rate is unknown by BSV, but can by definition be assumed to be 0%), a 15% increase in screening (power=80% and Type 1 error rate=5%) would require a total pooled sample size of 200. Given that the number of Italian women is approximately 30% higher, this sample size can be allocated as:

- Arabic women=43 in each arm. This will detect a 25% increase in screening rates.
- Italian women=57 in each arm. This will detect between 20-25% increase in screening rates.

These sample sizes are feasible. Between May and October 2017 in the North-west Melbourne region there are n=1,376 Italian women and n=525 Arabic women due for their 2-yearly screen, and in 2014 (the latest available data), there were n=563 Italian and n=243 Arabic women who were lapsed screeners.

Analysis: Analysis will be on an intention-to-treat basis. This is required, as Group 2 (routine reminder letters and phone calls in language) will not all require a follow-up phone call if they book a screening appointment within 2 weeks of the routine reminder letter being posted. For each cultural group, categorical data will be presented as frequencies and percentages. No continuous data will be collected for this study. Primary and secondary outcomes will be modelled as proportions. Differences between control and intervention groups will be analysed using chi-square. Where feasible, sub-group analysis will be undertaken by screening site.

Figure 1: Sample size and allocation

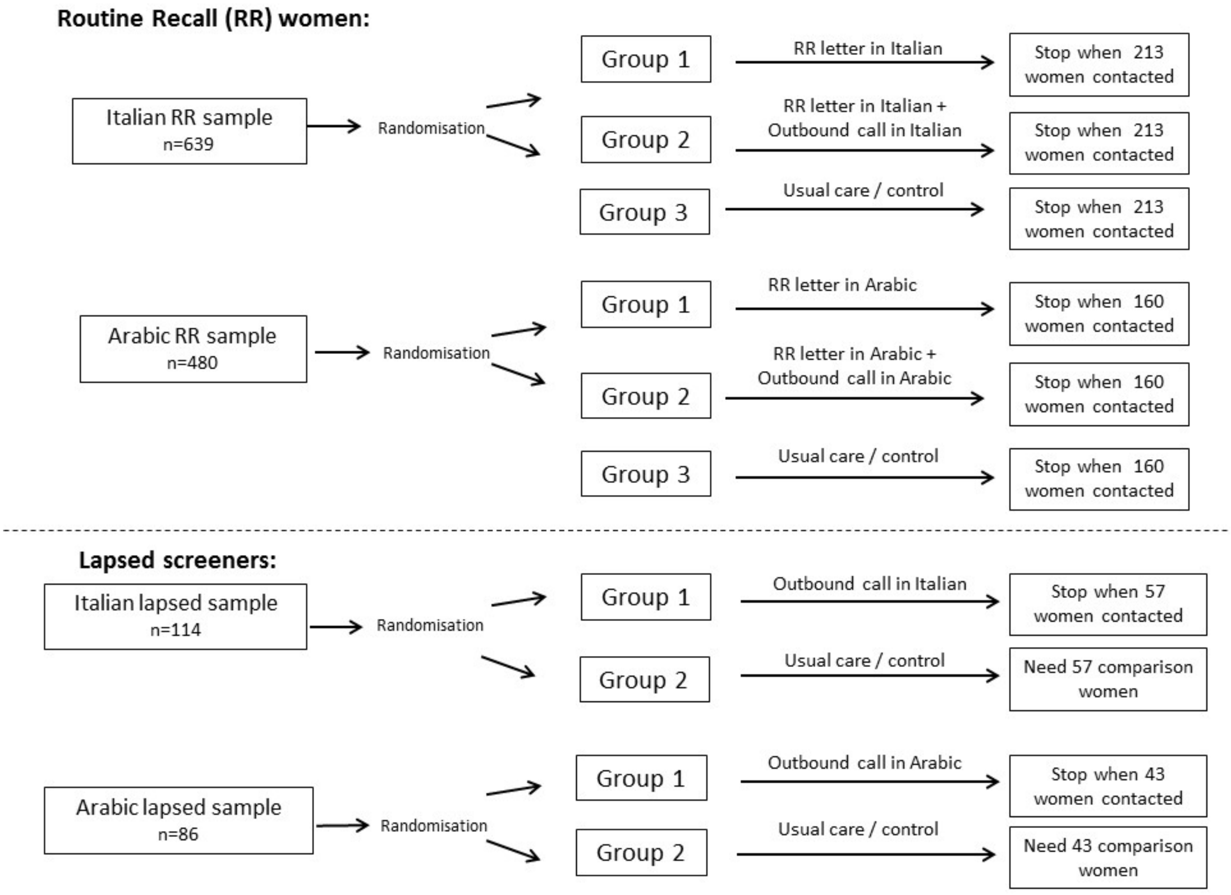

Dissemination of findings: It is planned that this service improvement initiative will be presented to DHHS and reported in peer-reviewed articles and presented at conferences.
