# Supplementary material for: The impact of translated reminder letters and phone calls on mammography screening booking rates: Two randomised controlled trials
Source: PLoS One. 2020 Jan 10;15(1):e0226610. doi: 10.1371/journal.pone.0226610 (PMC6953872; doi:10.1371/journal.pone.0226610)
Supplement: S2 Protocol — (PDF) [file pone.0226610.s006.pdf]

Overview of study protocol and ethics documents

- Ethics approval was sought as an **amendment to** a larger Breast Screen Ophelia study within which these RCTs were conducted (see '1. Study Protocol', which describes this amendment). Please note, this Study Protocol initially included 3 study arms, with the third arm receiving both a letter and a phone call. This Study Protocol was approved by Deakin University Human Research Ethics Committee (DUHREC) as an amendment on 22/05/2017.
- Prior to registration of the trial with ANZCTR, BSV informed us that due to an overestimation of the number of women who were forecast to receive their 2-yearly reminder letter, they considered that it was not achievable to also include the third arm (letters and phone calls). This group was therefore dropped from the trial, and was not included in the information provided to ANZCTR. The trial was submitted to ANZCTR on 28/07/2017 and registered on 8/8/17.
- We notified DUHREC that the third arm of the trial was not included (see 2. Ethics Modification_Application and protocol). This modification was approved on 16/1/18.
- The original protocol also includes a secondary outcome being the source of referral as self-reported by women when booking a screening appointment; however, this data was never collected as BreastScreen Victoria decided the data were unlikely to be reliable.
